# Progress in Neurology

**Published:** 1903-01-17

**Authors:** 


### PROGRESS IN NEUROLOGY.

(*Concluded from page* 250.)

**Spastic Spinal Paralysis.**—Erb,[3] the discoverer of spastic spinal paralysis, has in a recent lecture described the history and characteristics of the disease. The disease, which is a very rare one, begins slowly with a sense of weight and feebleness in the legs, and without any other symptom than backache. The condition progresses just as slowly as it commenced, the legs becoming feebler and stiffer. There is on objective examination a more or less marked spastic contracture of the muscles of the legs, a certain degree of weakness which hardly ever amounts to actual paralysis, and exaggeration of the tendon jerks. To this must be added the extensor plantar reflex, discovered by Babinski. There are no disturbances of sensation, no Romberg's sign, no change in the functions of the bladder, bowel, or generative organs, no muscular atrophy, no disturbances of the functions of the brain. The disease progresses as slowly as it began—endlessly for years and decades; it is not directly fatal, and there is less distress and suffering than in most of the chronic progressive spinal affections. The picture of the family or hereditary form of the disease, occurring in members of the same family and in several generations, is essentially the same. The anatomical lesion is a degeneration of the lateral tracts in the spinal cord, which though not always confined to these tracts is most strongly marked in them. The only difficulty in diagnosis is to separate the affection from multiple sclerosis, in which the four cardinal symptoms mentioned above may exist alone. The differential diagnosis may be for a time impossible, but in multiple sclerosis other symptoms—such as nystagmus, tremors, bladder trouble—are soon added. Syphilitic spinal paralysis is an affection, also first differentiated by Erb, which has a close resemblance to spastic spinal paralysis. Briefly, it is characterised by this, that besides the typical picture of spastic spinal paralysis, there are always a disturbance in bladder function, and usually a slight objective and subjective disturbance of sensation. The rigidity of the muscles, however, is slight when compared with the very spastic gait. The onset of the disease is usually insidious, sometimes more rapid, often from two to six years after the syphilitic infection. The pathological change in the cord is a degeneration of the lateral pyramidal tracts, the cerebellar tracts, and the posterior columns. To the disease in the posterior columns are to be referred the disturbances in sensation and the bladder trouble. The disease is to be distinguished from simple spastic paralysis by the disturbances of sensation and bladder function; from transverse myelitis by the absence of marked paralytic phenomena, and from meningo-myelitis by the absence of meningitic and root symptoms. The prognosis is not so unsatisfactory as in the case of simple spastic paralysis—under energetic mercurial treatment considerable improvement often takes place, though complete recovery is rare.

**Epilepsy.**—Voison's[4] experience goes to show that epileptic seizures are generally modified for the better by the following specific diseases: erysipelas, anthrax, acute pneumonia, measles, phthisis, and diphtheria. The attacks ceased during these illnesses, but if they were recovered from, soon resumed their original frequency and violence. The effect, then, was only transient. On the other hand, scarlatina, influenza, and typhoid fever generally increase the frequency of the attacks. These different results of different specific fevers show that the raised temperature is not of itself the cause of the temporary modifications of the disorder; the result must be due to the specific toxins of the intercurrent diseases. McCarthy recommends the use of chloretone, where bromides have failed or given rise to objectionable symptoms, such as mental torpor, or lack of concentration; in such cases 15 gr. of chloretone every night is sometimes attended with excellent results, as far as the fits are concerned, with but little, if any, depressing effects. Chloretone is of no use in cases where the attacks begin early in life and are associated with dementia. In status epilepticus

Hoppe has used Dormiol with good results; in half to one hour the convulsions ceased, and the patients fell into a prolonged sleep. The drug was given by rectal injection, the dose being 35 gr.

Browning[5] says that in young children epileptiform attacks, of various types, may occur, which are due to or associated with disturbances in the general tissue metabolism of the body. Some of these are in whole or in part of rachitic origin. He has found that when troubles of this kind occur they are amenable to thyroid treatment. On the other hand, true epilepsy is not remedied by thyroid, even in a person who was once rachitic. It seems that in many cases there is a closer relationship between rachitis and athyreosis than has been recognised—there must be a relative inadequacy of the thyroid function in these cases associated with rickets.

Spratling[6] is of opinion that bromipin, a brominized oil of sesamum, which has lately come into use, is a good substitute for the bromides in epilepsy. It is best given in the form of an emulsion; the following is a good formula:—

Bromipin, oz. 2.

Yolks of 2 eggs.

Menthol, gr. 2½.

(or Spt. Menth. pip. ♏ 30).

Brandy, oz. ½. M. S. ʒi. ter die.

The advantages of bromipin are that it does not produce any disfiguring bromide acne, and given in the form of an emulsion it is nutritious. It may further be given hypodermically in status epilepticus without producing local abscesses, such as may follow the hypodermic administration of bromides. In status patients, the best formula for stopping the attacks is the following:—

℞ Pot. Bromid, oz. 2.

Chloral hydrat, ʒ 5.

Morph. sulphat, gr. 2.

Tinct. Opii, ♏ 60.

Aq., ad ℥ xvi. M.

Give the patient 1 oz. after he has had six attacks in rapid succession. If the first dose is not effective, it may be repeated in two hours.

Ceni[7] claims to have produced remarkable results in the treatment of epilepsy by the injection of blood serum taken from other patients also suffering from epilepsy. The results were very good, the fits were much reduced in number, and there was a great improvement in the bodily and mental processes. Russell,[8] in criticising this paper, points out that it is difficult to understand how the injection of serum containing small quantities of harmful products with which the organism has long been poisoned could have any beneficial effect. Further, on examining the detailed reports of the cases, it is clear that the patients treated had been previously saturated with bromides for long periods of time, and that on the introduction of the serum treatment the bromides were left off. It is therefore very probable that the amelioration in the condition of the patients was due simply to the omission of the bromides.

**Chorea.**—Pope,[9] from a long experience, finds that in a large majority of cases of chorea a cure can be effected in from seven to ten days by the administration of liq. arsenicalis in rapidly increasing doses. The treatment is inadvisable in very acute cases with coma or paralysis, in those who have been treated for some time with small doses of arsenic, in cases where there is reason to think that the rheumatic processes are still going on in an acute or subacute form, and where there is advanced cardiac disease. The principles of administration are: the tongue should be clean, if it is not a mild mercurial purge, and a stomachic mixture should be first given. Put the patient on a bland diet, mostly consisting of milk. Give the drug in a much diluted form and in the same dilution throughout, *e.g.*, 2½♏ of liq. arsenicalis in 1 oz. of water three times a day after food. Each day increase each dose by 1 oz., so that, for instance, on the fourth day the patient is taking 4 oz. three times in the 24 hours. Do not discontinue on the first attack of vomiting, which may be due to accidental causes; if the vomiting persists, discontinue the drug for 24 hours. Examine the patient very carefully daily for any sign of toxic action. By this means we get the greatest immediate action with the least risk of doing permanent injury. It is found that in seven or eight days the disease is either entirely controlled or becomes but trifling.

[3] Lancet, October 11. [4] Birm. Med. Rev., May. [5] Jour. of Nerve and Ment. Dis., October. [6] Jour. Amer. Assoc., May 3. [7] Med. News, March 8 and 15. [8] Birm. Med. Rev., September. [9] Brit. Med. Jour., October 18.

